# Genomic Analysis Identifies Mutations Concerning Drug-Resistance and Beijing Genotype in Multidrug-Resistant *Mycobacterium tuberculosis* Isolated From China

**DOI:** 10.3389/fmicb.2020.01444

**Published:** 2020-07-15

**Authors:** Li Wan, Haican Liu, Machao Li, Yi Jiang, Xiuqin Zhao, Zhiguang Liu, Kanglin Wan, Guilian Li, Cha-xiang Guan

**Affiliations:** ^1^Department of Physiology, Xiangya School of Medicine, Central South University, Changsha, China; ^2^State Key Laboratory for Infectious Disease Prevention and Control, Collaborative Innovation Center for Diagnosis and Treatment of Infectious Diseases, National Institute for Communicable Disease Control and Prevention, Chinese Center for Disease Control and Prevention, Beijing, China

**Keywords:** *Mycobacterium tuberculosis*, resistance, whole-genome sequencing, mutation, Beijing genotype

## Abstract

Development of modern genomics provides us an effective method to understand the molecular mechanism of drug resistance and diagnose drug-resistant *Mycobacterium tuberculosis*. In this study, mutations in 18 genes or intergenic regions acquired by whole-genome sequencing (WGS) of 183 clinical *M. tuberculosis* strains, including 137 multidrug-resistant and 46 pan-susceptible isolates from China, were identified and used to analyze their associations with resistance of isoniazid, rifampin, ethambutol, and streptomycin. Using the proportional method as the gold standard method, the accuracy values of WGS to predict resistance were calculated. The association between synonymous or lineage definition mutations with different genotypes were also analyzed. The results show that, compared to the phenotypic proportional method, the sensitivity and specificity of WGS for resistance detection were 94.2 and 100.0% for rifampicin (based on mutations in *rpoB*), 90.5 and 97.8% for isoniazid (*katG*), 83.0 and 97.8% for streptomycin (*rpsL* combined with *rrs* 530 loop and 912 loop), and 90.9 and 65.1% for ethambutol (*embB*), respectively. WGS data also showed that mutations in the *inhA* promoter increased only 2.2% sensitivity for INH based on mutations in *katG*. Synonymous mutation *rpoB* A1075A was confirmed to be associated with the Beijing genotype. This study confirmed that mutations in *rpoB*, *katG*, *rrs* 530 loop and 912 loop, and *rpsL* were excellent biomarkers for predicting rifampicin, isoniazid, and streptomycin resistance, respectively, and provided clues in clarifying the drug-resistance mechanism of *M. tuberculosis* isolates from China.

## Introduction

The World Health Organization’s (WHO’s) target is to end the tuberculosis epidemic by 2035 (Treatment Action Group and Stop Tb Partnership, 2018). The evolution and spread of rifampicin-/multidrug-resistant tuberculosis (RR-TB/MDR-TB) poses a major obstacle to success with an estimated half a million cases worldwide in 2018 alone (United Nations General Assembly, 2018). WHO estimated that only one in three of the approximately half a million RR-TB/MDR-TB cases were enrolled in treatment with a second-line regimen (World Health Organization, 2019). Closing the gap in detection and treatment of resistant TB cases requires much higher coverage of drug susceptibility testing among people diagnosed with TB and rapid, accurate, and sensitive susceptibility testing methods.

Generally, the culture-based conventional drug sensitivity test (DST) has long been considered as the gold standard for diagnosing drug-resistant *Mycobacterium tuberculosis* although it is time-consuming, labor-intensive, and lacking sensitivity. Therefore, nucleic acid–based antibiotic susceptibility tests, which can determine the isolate’s drug resistance profile within hours instead of culture-based diagnostics that require days or weeks, are increasingly considered as a diagnostic alternative. Acquired antibiotic resistance in *M. tuberculosis* mostly arises from the serial acquisition of point mutations in genes encoding drug targets or drug-activating enzymes. Many known mutations have been identified, e.g., *katG*315 and *inhA*(-15) for isoniazid (INH) resistance (Liu et al., 2018; Luo et al., 2019; Unissa et al., 2016), 81-bp of rifampicin (RMP) resistant determined region (RRDR) for RMP resistance (Li et al., 2010; Maningi et al., 2018; Luo et al., 2019), *embB*306 for ethambutol (EMB) resistance (Mokrousov et al., 2002; Campbell et al., 2011; Moure et al., 2014; Zhao et al., 2015a), and *rpsL*44 and *rpsL*88 for streptomycin (STR) resistance (Li et al., 2010; Maningi et al., 2018). Current molecular diagnostics amplify and detect known drug resistance–associated mutations, and their performance depends on the inclusion of a comprehensive catalog of these mutations. Although known mutations explain much resistance in *M. tuberculosis*, causative mutations have not been identified in 10–40% of clinically resistant isolates (Campbell et al., 2011; Zhang et al., 2014; Zhao et al., 2014a), implying the contribution of noncanonical mutations in known or unknown genes or other resistance mechanism(s). Mutations outside of RRDR in *rpoB* or mutations except the canonical mutation *katG*315 in *katG* have been reported to be associated with RMP or INH resistance, respectively (Zhao et al., 2014b; Torres et al., 2015). Maningi et al. (2018) reports that whole-genome sequencing (WGS) shows better concordance with the Lowenstein–Jensen (L-J) phenotypic assay than with Hain line probe assay in that many more mutations were found by WGS. Moreover, many genes, such as *ndh*, *efpA*, *kasA*, *iniABC* operon (for INH resistance) (Sandgren et al., 2009; Nguyen, 2016); *rpoC* (RMP) (Farhat et al., 2013; Perdigao et al., 2020); *embA*, *embC*, *ubiA* (EMB) (Plinke et al., 2010; Zhao et al., 2015a; Farhat et al., 2016); and *gidB* (STR) (Nhu et al., 2012; Spies et al., 2011; Perdigao et al., 2014), are reported to correlate with drug resistance.

Whole-genome sequencing enables the screening of known resistance-associated loci while also providing opportunities to characterize other loci as predictive of resistance or not (Farhat et al., 2013; Casali et al., 2014). In this study, we analyze the sequence polymorphisms in 18 chosen genes or regions of 183 *M. tuberculosis* isolates based on the whole-genome sequenced data. The sequences or regions were chosen on the basis of their demonstrated association with drug resistance and according to the TB Drug Resistance Database (Sandgren et al., 2009).

## Materials and Methods

### Strains

We used 183 *M. tuberculosis* isolates for WGS. H37Rv (ATCC 27294), which is susceptible to the four first-line anti-tuberculosis drugs INH, RMP, EMB, and pyrazinamide (i.e., pansusceptible), was used as a reference. The isolates used for WGS were obtained from 183 adult patients with pulmonary TB from 2005 to 2009 from institutes for tuberculosis control and prevention as well as tuberculosis hospitals distributed in 11 provincial-level administration divisions (PLADs) of China; the numbers isolated from each PLAD were as follows: Beijing, 13; Fujian, 24; Guangdong, 8; Guizhou, 21; Henan, 6; Inner Mongolia, 5; Liaoning, 20; Shaanxi, 25; Shanghai, 26; Tibet, 30; and Xinjiang, 5.

### Drug Susceptibility Testing and Mycobacterium Species Identification

The isolate profiles of drug susceptibility were evaluated in our laboratory by the proportional method using L-J slants with the following drug concentrations: INH, 0.2 μg/mL; RMP, 40 μg/mL; STR, 4 μg/mL; EMB, 2 μg/mL; kanamycin (KAN), 30 μg/mL; ofloxacin (OFX), 2 μg/mL; capreomycin (CAP), 40 μg/mL (World Health Organization, 2008). L-J medium containing para-nitrobenzoic acid (500 μg/mL) was used to identify *M. tuberculosis* complex species from non-tuberculosis mycobacteria, and medium containing thiophen-2-carboxylic acid hydrazide (5 μg/mL) was used to exclude *Mycobacterium bovis* (*M. bovis*) from the *M. tuberculosis* complex. This study included the *M. tuberculosis* complex but did not include *M. bovis* clinical isolates. All the strains were stored in physiological saline containing 50% glycerol at −70°C. Prior to characterizing the drug susceptibility, the strains were recovered on L-J medium for 4 weeks at 37°C. DST, mycobacterium species identification, and inactivation of strains were all performed in a Biosafety Level 2 Laboratory.

### Genome Sequencing

Genomic DNA was extracted from *M. tuberculosis* colonies on L-J medium using CTAB (van Embden et al., 1993). DNA libraries (350 bp insert) were constructed with genomic DNA using kits provided by Illumina according to the manufacturer’s instructions. DNA libraries were then selected to perform cluster growth and 90 bp paired-end sequencing on an Illumina HiSeq 2000 sequencer according to standard protocols. Raw reads with consecutive bases covered by fewer than five reads, duplicate reads, and the adapter were removed; then, the rest of the reads, called clean data, from each strain were mapped to the genome of H37Rv (GenBank accession number, NC_000962.2) using SOAP2 (Li et al., 2009). An average of 5.27 million sequence reads were acquired per genome at a depth of 112× and with coverage of 99.32%. The accuracy of the sequencing was assessed by sequencing *rpoB* in a random selection of 80 isolates on an ABI Prism 3730 automated DNA sequencer (ABI, Shirley, NY, United States) as described by Zhang et al. (2013), the results show 100% consensus between the Illumina HiSeq 2000 and ABI 3730 sequencing results.

### Identify Mutations in Drug Resistance–Associated Genes and Promoter Regions

Identification of resistance-causing single-nucleotide polymorphisms (SNPs) from genome-wide sequence is challenging. We focused on putative or known resistance genes and promoter regions on the basis of their demonstrated association with drug resistance and according to the TB Drug Resistance Database (Sandgren et al., 2009; Table 1). All mutations in these genes and promoter regions were compared with the pan-susceptible reference genome (H37Rv, accession number: NC_000962.2) at the level of SNPs in promoter regions, amino acids in genes, or insertions and deletions. In this study, we first characterized the synonymous and lineage-defining mutations that did not cause resistance, and then, we characterized the mutations associated with drug resistance. The phenotypic and genotypic results were compared to determine the specificity and sensitivity for each gene or region studied.

**TABLE 1:** Eighteen candidate genes and intergenic regions linked with acquisition of drug resistance.

Drug	Genes or regions	Product
Isoniazid	*katG*	Catalase-peroxidase-peroxynitritase
	*inhA* coding region	NADH-dependent enoyl-acyl carrier protein reductase
	promoter of *inhA*	–
	*ahpC* coding region	Alkyl hydroperoxide reductase C
	*oxyR-ahpC* intergenic region	–
	*ndh*	NADH dehydrogenase
	*efpA*	EfpA
	*kasA*	Beta-ketoacyl-ACP synthase
	*iniA*	Rv0342
	*iniC*	Rv0343
	*iniB*	Lipoprotein LpqJ
Rifampicin	*rpoB*	DNA-directed RNA polymerase β chain
Streptomycin	*rpsL*	30S ribosomal protein S12
	*rrs*	16S rRNA
	*gidB*	Glucose-inhibited division protein B
Ethambutol	*embB*	Arabinosyltransferase B
	*embC*	Arabinosyltransferase C
	*embA*	Arabinosyltransferase A

### Spoligotyping and Data Analysis

Spoligotyping was performed using 43 covalently bound oligonucleotides derived from the spacer sequences of *M. tuberculosis* H37Rv and *M. bovis* BCG P3 as previously described by Kamerbeek et al. (1997). The results in binary format were entered into an Excel spreadsheet and compared with the spoligotyping database SpolDB4.^1^

### Statistical Analysis

SPSS 16.0 (SPSS Inc., Chicago, IL, Untied States) was used to perform Chi-square and Fisher’s exact analysis according to the sample number and multivariate regression analysis. The difference was considered to be statistically significant when *P* < 0.05.

## Results

### Drug Susceptibility Patterns

All isolates for WGS underwent culture-based DST to seven drugs: 46 were fully drug susceptible; 137 were resistant to both INH and RMP. Among 137 MDR strains, 100 (73.0%), 77 (56.2%), 60 (43.8%), 22 (16.1%), and 21 (15.3%) were resistant to STR, EMB, OFX, KAN, and CAP, respectively; 18% (24/137) of the MDR isolates were extensively drug resistant (XDR, MDR isolates are also resistant to both fluoroquinolone and an injectable drug). Detailed susceptibility profiles are shown in Supplementary Table 1.

### Genotype Distribution of the *M. tuberculosis* Isolates

Among the *M. tuberculosis* isolates for WGS, 141 (77.0%) belonged to the Beijing genotype, and 42 (23.0%) were non-Beijing family, which included the T1 family (12), H3 family (3), T2 family (3), CAS family (2), Haarlem3 family (2), MANU2 family (2), CAS1-DELH1 family (1), LAM9 family (1), U family (1), and a new found genotype (12).

### Synonymous Mutations in Chosen Genes and Regions

A total of 55 synonymous mutations were found and are shown in Supplementary Table 2. Although synonymous mutations were universally acknowledged to be unrelated with drug resistance, we found that the prevalence of *rpoB* 1075 GCT-GCC (Ala-Ala) in RMP-resistant *M. tuberculosis* was higher than that in RMP-susceptible isolates, and the prevalence of *gidB* 205 GCA-GCG (Ala-Ala) in STR-resistant isolates was higher than that in STR-susceptible isolates. Among 55 synonymous mutations, 17 were found only in interested drug–susceptible isolates, 27 were found only in interested drug–resistant isolates, and the remaining 11 were found in both interested drug–susceptible and –resistant isolates. Sixteen out of the 27 synonymous mutations were found in the 11 genes or regions related with INH resistance in INH-resistant isolates. None of the stand-alone synonymous mutations in the known genes *katG* (INH), *rpoB* (RMP), and *rpsL* (STR) was found only in isolates resistant to INH, RMP, and STR, respectively, and one stand-alone synonymous mutation 304 CTG-TTG (L-L) in the known gene *embB* was only found in one EMB-resistant isolate.

We further analyzed the associations between the 55 synonymous mutations and the Beijing genotype and found that the prevalence of *iniA* 178 GGT-GGC (Gly-Gly) and *embA* 1092 GCG-GCA (Ala-Ala) was much higher in the non-Beijing genotype than in the Beijing genotype *M. tuberculosis*, and the prevalence of *rpoB* 1075 GCT-GCC (Ala-Ala), *embA* 76 TGC-TGT (Cys-Cys), and *gidB* 205 GCA-GCG (Ala-Ala) was much higher in the Beijing genotype than in the non-Beijing genotype. Among 55 synonymous mutations, 31 were only found in Beijing genotype strains, 17 were only found in the non-Beijing genotype, and seven were found in both genotypes. The results are shown in Supplementary Table 3.

We then performed multivariate analysis toward the mutations of *rpoB* 1075 GCT-GCC (Ala-Ala) and *gidB* 205 GCA-GCG (Ala-Ala) based on the univariate analysis. As shown in Table 2, the analysis data revealed that the Beijing genotype is the high-risk factor for these two synonymous mutations, not the RMP or STR resistance.

**TABLE 2:** Multivariate analysis of drug resistance and genotypes of *M. tuberculosis* according to the four mutations.

Dependent variables (mutations)	Characteristic	Adjusted ORs (95% CI)	*P-*value
*rpoB* 1075 GCT-GCC (Ala-Ala)	Beijing genotype	477.6 (110.2–2069.8)	0.000
	RMP resistance	0.4 (0.1–1.9)	0.266
*katG* 463 CGG-CTG (Arg-Leu)	Beijing genotype	477.6 (110.2–2069.8)	0.000
	INH resistance	0.4 (0.1–1.9)	0.266
*gidB* 92 GAA-GAC (Glu-Asp)	Beijing genotype	453.2 (116.9–1757.5)	0.000
	STR resistance	1.6 (0.4–6.0)	0.523
*gidB* 205 GCA-GCG (Ala-Ala)	Beijing genotype	334.1 (90.8–1229.4)	0.000
	STR resistance	1.1 (0.3–4.0)	0.859

*RMP, rifampicin; STR, streptomycin; INH, isoniazid.*

### Analysis on Mutations *katG* R463L and *gidB* E92D Known Not to Code for Resistance

In the present study, 146 and 143 out of 183 isolates carried mutations *katG* 463 CGG-CTG (Arg-Leu) and *gidB* 92 GAA-GAC (Glu-Asp), respectively. According to previous studies, both mutations are known not to be associated with resistance (Meacci et al., 2005; Via et al., 2010; Feuerriegel et al., 2014; Nebenzahl-Guimaraes et al., 2014), and *gidB*92 polymorphism (276C allele) has been reported to be associated with the Beijing lineage (Spies et al., 2011). However, statistical analysis shows that the prevalence of *katG* 463 CGG-CTG (Arg-Leu) in INH-resistant isolates are higher than that in INH-susceptible isolates, and the prevalence of *gidB* 92 GAA-GAC (Glu-Asp) in STR-resistant isolates is higher than that in STR-susceptible isolates; both *P*-values were less than 0.05 (see Supplementary Table 4). We also found that the prevalence of *katG* 463 CGG-CTG (Arg-Leu) and *gidB* 92 GAA-GAC (Glu-Asp) in the Beijing genotype is much higher than in the non-Beijing genotype isolates; see Supplementary Table 5.

Multivariate analysis toward the mutations of *katG* 463 CGG-CTG (Arg-Leu) and *gidB* 92 GAA-GAC (Glu-Asp) based on the univariate analysis shows that the Beijing genotype is the high-risk factor for both mutations, not the INH resistance or STR resistance (Table 2).

### Drug Resistance and Gene Mutations

#### Isoniazid Resistance and Mutations in Genes and Intergenic Regions

**FIGURE 1:**
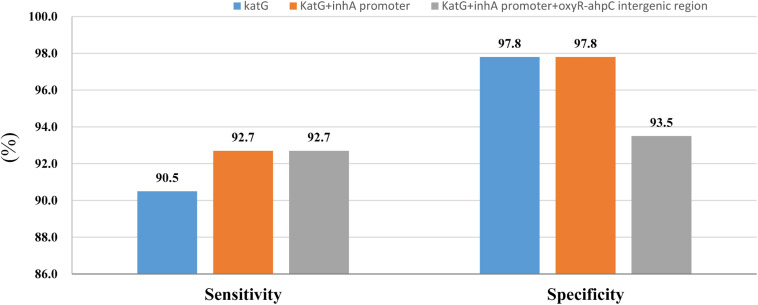
Sensitivity and specificity of sequencing of *katG* combined with *inhA* promoter and *oxyR-ahpC* intergenic region for isoniazid resistance and susceptibility diagnoses.

**FIGURE 2:**
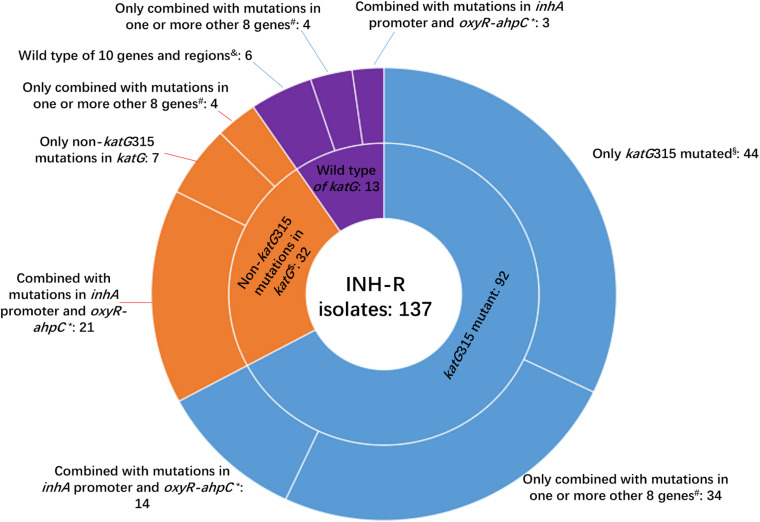
Number of isolates carrying *katG* mutations combined with mutations in another 10 genes or intergenic regions among 137 MDR *M. tuberculosis*. INH-R, isoniazid resistant; *this group included isoniazid-resistant isolates that also carried mutations in *ahpC* coding region, *inhA* coding region, *ndh*, *efpA*, *kasA*, *iniA*, *iniB*, and/or *iniC*. ^#^Eight genes included *ahpC* coding region, *inhA* coding region, *ndh*, *efpA*, *kasA*, *iniA*, *iniB*, and *iniC*; ^$^two isolates that had mutations of *katG*315 and other substitutes in *katG* were not included in this group. **^§^** This group included two isolates that had mutations of *katG*315 and other substitutes in *katG*; ^&^10 genes and regions included *inhA* promoter, *oxyR*-*ahpC* intergenic region, *ahpC* coding region, *inhA* coding region, *ndh*, *efpA*, *kasA*, *iniA*, *iniB*, and *iniC.*

Whole-genome sequencing data shows that, respectively, among 137 INH-resistant and 46 INH-susceptible isolates, 124 and 1 carry mutations in *katG*, 28 and 0 in the *inhA* promoter, and 12 and 2 in the *oxyR-ahpC* intergenic region (Table 3). We found a rare high prevalence (90.5%) of the *katG* mutation in INH-resistant *M. tuberculosis*, which has never been reported in China. *katG* mutations combined with that in the *inhA* promoter only increased the sensitivity from 90.5 to 92.7% while there was no additional specificity (Figure 1). At the base of mutations in *katG* and the *inhA* promoter, adding the mutations in the *oxyR-ahpC* intergenic region, the sensitivity was not changed although the specificity fell from 97.8 to 93.5% as shown in Figure 1. The most frequent mutation site was *katG*315 (92/137, 67.2%); among the isolates that carried this mutation, only 14 combined mutations in the *inhA* promoter and/or the *oxyR-ahpC* intergenic region; among 32 INH-resistant isolates, which carried mutations in *katG* non-315, 21 combined mutations in the *inhA* promoter and/or the *oxyR-ahpC* intergenic region; among 13 INH-resistant isolates, which carried the wild-type of *katG*, three carried mutations in the *inhA* promoter and/or the *oxyR-ahpC* intergenic region, four carried mutations only in the other eight genes related to INH resistance, and there were still six INH-resistant strains with the wild-type of 11 sequenced genes that have been reported to be associated with INH resistance; see Figure 2. Among 28 INH-resistant isolates that carried mutations in the *inhA* promoter, 11 carried mutations of *katG*315, 14 carried mutations in *katG* but not in codon 315, and three carried wild-type *katG*.

**TABLE 3:** The evaluation between whole-genome sequencing analysis of 18 drug-resistant associated genes or regions and the phenotypic drug susceptibility testing.

Drugs	Genes	Number of isolates (%*) carried mutations^&^ in resistant strains	Number of isolates (%^#^) carried mutations^&^ in susceptible strains	χ^2^	*P*	Sensitivity^a^ (%)	Specificity^b^ (%)
INH	*katG*	124 (90.5)	1 (2.2)	119.2	0.000	90.5	97.8
	*inhA* promoter	28 (20.4)	0 (0.0)	11.1	0.001	20.4	100.0
	*OxyR-ahpC* intergenic region	12 (8.8)	2 (4.3)	0.4	0.514	8.8	95.7
	*inhA* coding region	3 (2.2)	0 (0.0)	0.1	0.733	2.2	100.0
	*ahpC* coding region	1 (0.7)	0 (0.0)	0.0	1.0	0.7	100.0
	*ndh*	18 (13.1)	5 (10.9)	0.2	0.688	13.1	89.1
	*kasA*	3 (2.2)	0 (0.0)	0.1	0.733	2.2	100.0
	*efpA*	3 (2.2)	0 (0.0)	0.1	0.733	2.2	100.0
	*iniA*	5 (3.6)	1 (2.2)	0.0	0.994	3.6	97.8
	*iniB*	32 (23.4)	12 (26.1)	0.1	0.708	23.4	73.9
	*iniC*	8 (5.8)	2 (4.3)	0.0	0.992	5.8	95.7
RMP	*rpoB*	129 (94.2)	0 (0.0)	146.8	0.000	94.2	100.0
STR	*rpsL*	72 (72.0)	3 (3.6)	87.70	0.000	72.0	96.4
	*rrs* 530 loop and 912 loop	11 (11.0)	0 (0.0)	7.86	0.005	11.0	100
	*gidB*	12 (12)	7 (8.4)	0.62	0.431	12.0	91.6
EMB	*embA*	7 (9.1)	9 (8.5)	0.02	0.887	9.1	91.5
	*embB*	70 (90.9)	37 (34.9)	57.61	0.000	90.9	65.1
	*embC*	4 (5.2)	9 (8.5)	0.73	0.392	5.2	91.5

INH, isoniazid; RMP, rifampicin; STR, streptomycin; EMB, ethambutol. *Compared with the total number of isolates resistant to the interested drug: INH, 137; RFP, 137; STR, 100; EMB, 77. ^&^Mutations used here did not include synonymous mutations and well-known nonsynonymous polymorphisms (*katG* R463L and *gidB* E92D) which universally acknowledged unrelated with drug resistance. ^#^Compared with the total number of isolates susceptible to the interested drug: INH, 46; RFP, 46; STR, 83; EMB, 106. ^*a*^Sensitivity = number of interested drug resistant isolates with mutation/total number of interested drug resistant isolates. ^*b*^Specificity = number of interested drug susceptible isolates without mutation/total number of interested drug susceptible isolates.

In the present study, a total of 29 mutation sites in *katG* except *katG315* were found in 34 INH-resistant isolates of which two were combined with the mutation of *katG*315 (Figure 2 and Supplementary Table 6). We also found that 23 out of 45 isolates possessed *katG* non-315 mutations, or wild-type *katG* carried *inhA* C(-15)T and/or mutations in the region of *ahpC* from −84 to −48, which occupied 16.8% of the INH-resistant isolates. So the *inhA*(-15) and *ahpC*−84 to −48 combined with *katG*315 can make a preferable set for INH-resistance diagnoses, and the mutation sites in *katG* except 315 were scattered, which made these codons have less diagnostic value when used in gene chip, line/dot-blot hybridization or multiplex fluorescence melting curve analyses.

Besides *katG*, the *inhA* promoter, and *oxyR-ahpC* intergenic region, eight genes (coding regions) were also included to clarify the mechanism of INH resistance. The strain numbers that carried mutations in eight studied genes (coding regions) in INH-resistant and -susceptible isolates were as follows, respectively: *inhA* coding region, 3 and 0; *ahpC* coding region, 1 and 0; *ndh*, 18 and 5; *kasA*, 3 and 0; *efpA*, 3 and 0; *iniA*, 5 and 1; *iniB*, 32 and 12; *iniC*, 8 and 2 (Table 3). Low mutation prevalence of these genes in INH-resistant strains and mutations has even been found in INH-susceptible strains, which made the associations confused between these genes and INH resistance.

As shown in Table 4, we found 18 novel mutations, which included 10 in the gene *katG*, three in the *oxyR-ahpC* intergenic region, one in the *ahpC* coding region, two in the *kasA* gene, and four in the *efpA* gene.

**TABLE 4:** Novel mutations occurred in genes and regions associated with isoniazid resistance and found only in phenotypic isoniazid resistant *M. tuberculosis*.

Genes	Codon change(s)	Amino acid/nucleotide changes	Combined mutations in *katG*, *inhA* promoter or *oxyR-ahpC* intergenic region	Number of INH resistant isolates*
*KatG*	TGG-AGG	Trp90Arg	*ahpC* G(-48)A	1
	CTG-CGG	Leu587Arg	*katG* Tyr155Cys and *ahpC* C(-72)T	1^a^
	GAC-GCC	Asp189Ala	none	1
	GAC-GGC	Asp419Gly	*katG* Pro232Ser	1
			*ahpC* (-47) insert T	1^b^
	CCG-TCG	Pro235Ser	*katG* Ser302Arg	1
	GCC-ACC	Ala649Thr	*katG* Ser315Thr	1
	ACT-CCT	Thr380Pro	*inhA* C(-15)T	1
	–	Nucleotide positions 861-866 deleted ACCCGA	None	1
	–	Nucleotide positions 86–88 deleted CC	*inhA* C(-15)T	1
	–	nucleotide position 956 deleted T	*ahpC* C(-81)T	1^c^
*oxyR-ahpC* intergenic region	–	(-72) C-T	*katG* Tyr155Cys and Leu587Arg	1^a^
			*katG* Gly169Ser and *inhA* C(-15)T	1
			*katG* Leu378Pro	1
	–	(-47) insert T	*katG* Asp419Gly	1^b^
	–	C(-81)T	nucleotide position 1735 deleted A in *katG*	1
			nucleotide position 956 deleted T in *katG*	1^c^
*ahpC* coding region	AGC-AGA	Ser148Arg	*katG* Ser315Thr and *inhA* C(-8)T	1
*kasA*	GTT-ATT	V142I	*katG* Ser315Thr	1
	CAC-TAC	H253Y	*katG* Ser315Thr	2
*efpA*	ACA-CCA	T7P	*katG* Ser315Thr	1^d^
	GCC-GAC	A227D	*katG* Ser315Thr	1^d^
	CTG-ATG	L275M	none	1
	ATC-GTC	I313V	*katG* Ser315Thr	1

*Identical letters in this column means that these mutations were occurred in the same isoniazid resistant isolates.

### Rifampicin Resistance and *rpoB* Mutations

The whole *rpoB* sequence of 183 *M. tuberculosis* was analyzed. Altogether, 94.2% (129/137) of the RMP-resistant isolates harbored at least one mutation within *rpoB*, and other eight isolates lacked such a mutation (Supplementary Table 7). Eighty-nine isolates (65%) had a single mutation, and 48 (35%) had two or more mutations each. When all of the mutations were considered, regardless of single, double, or more, a total of 55 genotype patterns were identified, and 127 out of 137 RMP-resistant *M. tuberculosis* isolates carried mutations in the 81-bp RRDR of the *rpoB gene*. The most frequently mutated codons were 450 (*Escherichia coli* 531), 445 (*E. coli* 526), and 435 (*E. coli* 516) with mutation frequencies of 51.1% (70/137 isolates), 23.4% (32/137 isolates), and 12.4% (17/137 isolates) (Table 5). An independent and novel mutation was detected in *rpoB*: 675 GGC-GAC (Gly-Glu), which was only found in an RMP-resistant isolate. In contrast, none of 46 susceptible isolates possessed a nonsynonymous mutation within the whole sequence of the *rpoB* gene.

**TABLE 5:** Mutation frequency of codons of *rpoB* 81-bp rifampicin resistance determined region among 137 MDR isolates from China.

*M. tuberculosis* H37Rv codon number	*E. coli* codon number	Number (%) of rifampicin resistant isolates	Number of isolates without other *rpoB* mutation	Number of isolates combined with other *rpoB* mutations*
450	531	70 (51.1)	56	14
445	526	32 (23.4)	13	19
435	516	17 (12.4)	6	11
452	533	8 (5.8)	3	5
430	511	6 (4.4)	0	6
428	509	3 (2.2)	0	3
441	522	2 (1.5)	1	1
427	508	1 (0.7)	0	1
429	510	1 (0.7)	0	1
431	512	1 (0.7)	0	1
437	518	1 (0.7)	0	1
438	519	1 (0.7)	0	1

*Other *rpoB* mutations included the mutations which were in or outside of the rifampicin resistance determined region of *rpoB.*

### Streptomycin Resistance and Mutations in *rpsL*, *rrs*, and *gidB*

Previous studies show that mutations in loop 530 and loop 912 of *rrs* are associated with STR resistance (Finken et al., 1993; Sreevatsan et al., 1996; Li et al., 2010), so we only analyzed the mutations in these two regions of *rrs* and the whole sequence of *rpsL* and *gidB* in this study.

The WGS data shows that 72, 11, and 12 out of 100 STR-resistant isolates carried mutations in *rpsL*, *rrs* 530 loop, or 912 loop and *gidB*, respectively; in contrast, 3, 0, and 7 out of 83 STR-susceptible isolates carried, respectively (Table 3). None of the STR-resistant isolates were found to carry mutations simultaneously in both *rpsL* and *rrs* 530 loop or 912 loop. The sensitivity and susceptibility of mutations in *rpsL* combined with *rrs* 530 loop and 912 loop were 83.0% (83/100) and 92.8% (77/83). We also found that, among 83 STR-susceptible isolates, eight carried mutations in *rrs* outside of the 530 loop or 912 loop: one carried a mutation with nucleotides in positions 334–344 deleted, four carried mutations with nucleotides in positions 388–394 deleted, one carried a mutation of 555 A-T, and two carried mutations with 846 C-T and 1017 G-C. Among these eight isolates, seven were susceptible to INH, RMP, STR, EMB, CPM, KAN, and OFX.

As shown in Table 6, for *rpsL*, the most frequently mutated codons were 43 and 88 with mutation frequencies of 50.0% (50/100 isolates) and 19.0% (19/100 isolates), respectively. For the 530 loop and 912 loop, the most frequently mutated positions were 515, 518, and 888 with mutation frequencies of 8.0% (8/100 isolates), 2.0% (2/100 isolates), and 1.0% (1/100 isolates), respectively. In the present study, no isolate was found to carry mutations in *rpsL* combined with *rrs* 530 loop or 912 loop although one STR-resistant isolate was found to carry mutations of *rpsL* Lys88Arg and *rrs* 38 G-A. The mutation results of *gidB* were confused as shown in Table 3 and Supplementary Table 8; both STR-resistant and susceptible isolates carried mutations, and the mutated codons were scattered throughout the gene.

**TABLE 6:** Mutation characterizations of *rpsL* and *rrs 530 loop and 912 loop* among 100 streptomycin resistant *M. tuberculosis* from China.

Genes	Mutations	Frequency (Number of isolates)	Relative frequency^a^ (%)
*rpsL*	43AAG-AGG(Lys-Arg)	53	53.0
	88AAG-AGG(Lys-Arg)	19	19.0
*rrs* 530 loop and 912 loop	515A-C	8	8.0
	518C-T	2	2.0
	888G-T	1	1.0

*^*a*^Compared with the total number of isolates resistant to streptomycin.*

### Ethambutol Resistance and Mutations in *embB, embA*, and *embC*

Known mechanisms of EMB resistance are caused by mutations in the *embCAB* operon, especially in *embB*. The whole sequences of *embA, embB*, and *embC* were analyzed in this study. The results show that, among 77 EMB-resistant isolates, there were 7, 70, and 4 carried mutations in *embA*, *embB*, and *embC*, respectively, and among 106 EMB-susceptible isolates, there were 9, 37, and 9 carried mutations in *embA*, *embB*, and *embC*, respectively (Table 3).

The most predominant mutation in *embB* occurred at codon 306 (45, 58.4%), where the codon ATG (Met) was replaced with GTG (Val, 26, 33.8%), ATA (Ile, 11, 14.3%), ATC (Ile, 4, 5.2%), CTG (Leu, 2, 2.6%), ATT (Ile, 1, 1.3%), and GTA (Val,1, 1.3%), respectively. Mutation of *embB* Gly406 was the next most predominant mutation (9, 11.7%). Among the EMB-susceptible isolates, mutations in Met306 and Gly406 were also the most predominant in *embB*. As shown in Supplementary Table 9, among the EMB-resistant isolates, all of the mutations in *embA* or *embC* combined with mutations in *embB* and the mutated codons scattered in *embA* and *embC*.

## Discussion

The present study is the first to comprehensively analyze the whole sequences of 18 drug resistance–associated genes or intergenic regions of 183 *M. tuberculosis* isolates, which include MDR-TB and XDR-TB isolates from 11 provinces of China. The results suggest that sequencing the whole sequence of four genes can now characterize profiles of resistance to INH, RMP, and STR with an acceptable degree of accuracy sufficient for clinical use. Furthermore, more drug resistance–associated loci and Beijing genotype–associated loci were found in our research.

Compared to the molecular assays of DST, such as line-probe arrays, PCR methods based on TaqMan probes, or melting curves (Boehme et al., 2010; Chen et al., 2014; Zhao et al., 2015b; World Health Organization, 2016; Makhado et al., 2018; Cao et al., 2019), WGS displays better performance to predict drug resistance according to the limited known mutations as well as a big catalog of mutations in various genes. However, among the mutations found by WGS, synonymous mutations, mutations of lineage markers, and some well-known nonsynonymous polymorphisms (e.g., *katG* R463L, gyrA S95T) (Feuerriegel et al., 2014; Zhao et al., 2014b), which are universally acknowledged to be unrelated with drug resistance, must be excluded before predicting resistance. In the present study, we found that both *gidB* E92D and *katG* R463L occurred in almost all of the Beijing genotype strains although they sparsely occurred in strains with CAS, MANU, and new genotypes. As reported by previous studies (Jagielski et al., 2014; Sun et al., 2016), we also found that the nonsynonymous mutations *gidB* E92D and the synonymous mutation *gidB* A205A were associated with the Beijing genotype. The synonymous mutations *rpoB* A1075A and *embA* C76C were associated with the Beijing genotype, and *iniA* G178G and *embB* A1092A were associated with the non-Beijing genotype, which were first reported by us. The remaining 50 synonymous mutations were not found to have statistics association with drug resistance or genotypes.

It is well known that the mechanism of action of INH, which has a simple chemical structure, is very complex, and several bactericidal strategies have been reported (Timmins and Deretic, 2006). Consequently, several genes in multiple biosynthetic networks and pathways involved in INH action have been reported to play a role in INH resistance (Unissa et al., 2016). Mutations in the *katG* gene are the major contributors for INH resistance, followed by *inhA*, *ahpC*, *kasA*, *ndh*, *iniABC*, *efpA*, *fadE*, *furA*, *Rv1592c*, and *Rv1772* (Unissa et al., 2016). In the present study, There were 90.5% INH-resistant isolates that carried mutations in *katG*, which was higher than the 86.2% reported by Liu et al. (2018) and the 74.5% reported by Luo et al. (2019). Liu et al. (2018) reported that 47.1% (16/34) INH-resistant isolates had *inhA* promoter mutations combined with a mutation in *katG*, which was far lower than the 89.3% (25/28) found in our study. One explanation may be that they only sequenced 518 bp of *katG* while we analyzed the whole sequence of *katG*. In the present study, 29 additional mutations except *katG*315 were found in *katG*, of which 10 were novel mutations, and only three novel mutations combined with *katG*315 or *inhA*(-15) mutations. All of these novel mutations were found only in phenotypic INH-resistant isolates, suggesting that these mutations were resistance-associated but needed to be further verified by site-directed mutagenesis or other experiments. Many non-*kat*G315 mutations in the *katG* gene, e.g., Y95C, P131T, D142G, A162V, T306P, Y64S, F483L, and A541D, have been confirmed causing INH resistance, and *katG* R385W and D387G did not play a role in INH resistance by in vitro mutagenesis experiments (Torres et al., 2015).

In the present study, the mutation rates in *inhA* and *ahpC* coding regions, *kasA* and *efpA* in INH-resistant isolates were low. To make sure these mutations are associated with INH resistance, more studies for the phenotypic effect of these mutations are required. Another four genes (*ndh*, *iniA*, *iniB*, and *iniC*) show similar mutation frequency in both INH-resistant and -susceptible *M. tuberculosis*. The total mutation frequency of *ndh*, *iniA*, *iniB*, and *iniC* were 12.6% (23/183), 3.3% (6/183), 24% (44/183), and 5.5% (10/183), respectively. The mutation rate of *ndh* in INH-resistant isolates was higher than that reported by Islam et al. (13.1 vs. 2.9%) (Islam et al., 2019). Many reports have shown that isolates with mutations in *iniABC* had mutations in other genes as well (Ramaswamy et al., 2003), which was similar to our findings. However, the high mutation frequency of *iniABC* among clinical isolates in our study has never been reported. Previous studies have shown that IniA, IniB, and IniC were proteins that can be induced by isoniazid (Colangeli et al., 2005; Unissa et al., 2016). We speculated that there was a joint mechanism between efflux pumps and acquired mutations, e.g., *iniABC* and *katG*, and the accumulation of mutations under the pressure of drug selection may contribute to the appearance of INH resistance. Such knowledge of other genes (apart from *katG*) aids in developing better means to diagnose and prevent the transmission of INH-resistant tuberculosis.

Previous studies show that mutations in RRDR of *rpoB* account for 90% or higher RMP resistance (Li et al., 2010; Zhao et al., 2014b; Luo et al., 2019). We found 92.7% of RMP-resistant isolates carried mutations in this area, which is concordant with previous studies (Li et al., 2010; Zhao et al., 2014b; Luo et al., 2019). Only two RMP isolates carried mutations outside of RRDR: one carried a novel independent mutation of 675 GGC-GAC (Gly-Glu); the other isolate carried two substitutions of 170 GTC-TTC (Val-Phe) and 920 ATG-GTG (Met-Val). The most common mutations of the *rpoB* gene were in codons 450 (*E. coli* 531), 435 (*E. coli* 526), and 445 (*E. coli* 516) (Table 5); the results are consistent with other studies performed in China and other countries that reported the same trends (Li et al., 2010; Maningi et al., 2018; Luo et al., 2019). However, data from WGS in the present study reveal that 40 additional mutations outside of the RRDR region of *rpoB* are found in RMP-resistant phenotypes, and most of them are shown in the form of joint mutations with codons in RRDR, which may explain why isolates carrying the same mutation patterns in RRDR show different minimum inhibitory concentrations against RMP. Mutations in *rpoB* cannot answer the 5.8% RMP resistance in the present study. Previous studies show that substitutions in *rpoC* were frequent in RMP-resistant isolates; however, most *rpoC* substitutions combined with mutations in *rpoB* and are recognized as a modification or compensation for the phenotypes of mutations in the *rpoB* (Farhat et al., 2013; Perdigao et al., 2020).

Resistance to STR is due mostly to mutations in the *rpsL*, followed by mutations in *rrs* 530 loop or 912 loop. Only two mutations, Lys43Arg and Lys88Arg in *rpsL*, were found in STR-resistant isolates, similar to the data from other areas in China (Li et al., 2010) and South Africa (Maningi et al., 2018). The most common mutations of the *rrs* were A515C, C518T, and G888T, which are located in the *rrs* 530 loop or 912 loop. Eight out of 83 STR-susceptible isolates carried mutations outside of the *rrs* 530 loop or 912 loop, suggesting that these mutations are not related to STR resistance. Recently, mutations in *gidB* were reported to cause STR resistance (Perdigao et al., 2014; Verma et al., 2014). *gidB* mutations were found in both STR-resistant and -susceptible strains in the present study, consistent with data from previous studies (Nhu et al., 2012; Spies et al., 2011). Among 17 STR-resistant isolates without mutations in the *rpsL* or *rrs* 530 loop or 912 loop, seven carried *gidB* mutations found in six codons, of which one mutation (K163stop codon) was found in two STR-resistant isolates, and one mutation (nucleotide 120 C deleted) was also found in three STR-susceptible isolates. The results suggest that mutations in *gidB* do not help to explain STR resistance in isolates without the *rpsL* or *rrs* 530 loop and 912 loop mutations. The overexpressed proteins in STR-resistant isolates identified by Sharma and Bisht (2017b) are assumed to be responsible for STR resistance; however, the corresponding genes were not analyzed in the present study.

Resistance to EMB is mostly attributed to mutations in codon 306 in *embB*, accounting for 48.3–70.6% resistant isolates (Mokrousov et al., 2002; Campbell et al., 2011; Moure et al., 2014; Zhao et al., 2015a). In the present study, 58.4% EMB-resistant isolates carried mutations of *embB*306, in line with previous studies (Mokrousov et al., 2002; Campbell et al., 2011; Moure et al., 2014; Zhao et al., 2015a). Mutations of *embB*406 were found in 11.7% EMB-resistant isolates. WGS data from the present study found 19 more mutations besides the two canonical mutations in 16 EMB-resistant isolates, of which four isolates had double noncanonical mutations and one combined with *embB* M306I. *embB* mutations were found in 90.9% EMB-resistant isolates while previous studies ranged from 38.2 to 89.9% (Li et al., 2010; Campbell et al., 2011; Moure et al., 2014; Zhao et al., 2015a). However, 34.9% of EMB-susceptible isolates also carried mutations in *embB*, which is much higher that a previous study (6.5%) (CRyPTIC Consortium and the 100, 000 Genomes Project et al., 2018). One explanation may be that previous studies showed that the conventional DST method for EMB resistance was an imperfect standard, particularly for isolates with *embB* mutations (Zhang et al., 2009; Walker et al., 2015; Schon et al., 2017). A previous study in our laboratory shows that, using the EMB concentration with 1.6 μg/mL instead of 2.0 μg/mL in L-J slants by the proportional method, 23 out of 28 EMB-susceptible isolates that carried *emb*306 mutations could be successfully recognized as EMB-resistant isolates while the susceptibility patterns of 26 EMB susceptible isolates with wild-type *embB* were not affected (Zhang et al., 2009). A recent study shows that *embB* mutations are also associated with INH resistance in EMB-susceptible isolates (Wan et al., 2020) and another study shows that the *embB* M306I and M306V mutations are significantly associated with INH resistance in both EMB-resistant and -susceptible strains (Farhat et al., 2016). We speculate that the ambiguous relationship between mutations in *embB* and EMB or INH resistance may also lower the specificity of *embB* for predicting EMB resistance.

Mutations in *embC* and *embA*, which are suggested to be involved in EMB resistance development (Plinke et al., 2010; Zhao et al., 2015a; Farhat et al., 2016) were also analyzed in the present study; all of the isolates carried mutations in *embC* or *embA* combined with mutations in *embB*, and the prevalence of mutations in these two genes among EMB-resistant and -susceptible isolates were comparable. Therefore, 9.1% EMB resistance cannot be explained by *embC*, *embA* and *embB* in the present study. Farhat et al. (2016) finds univariate associations between *embA* N54D or *iniB* A70T and EMB resistance; however, no isolates carried these two mutations in the present study. Zhao et al. (2015a) reports that the mutations in the *embA* upstream region showed significant correlation with EMB resistance; however, this region was not included in the present study. Mutations in *ubiA* except a lineage-specific mutation E149D are reported to correlate with high-level EMB resistance and responsible for 3.2–6.4% EMB resistance (He et al., 2015; Xu et al., 2015; Tulyaprawat et al., 2019).

The WHO target product profiles for new molecular assays for *M. tuberculosis* require more than 90% sensitivity and 95% specificity (World health organization, 2014). Our findings show the predicted resistance to rifampicin and isoniazid exceeded 90% sensitivity and 95% specificity by WGS and analyzing *rpoB* (RMP) and *katG* (INH) (Table 3). In the present study, the additional mutation loci found in *katG* except in codon 315 made the mutations in the *inhA* promoter and *oxyR-ahpC* less meaningful for predicting INH resistance for that mutation in the latter two regions increased only 2.7% sensitivity by WGS. Although several molecular DSTs for INH resistance testing are recommended by WHO, several reports show that the calculated sensitivity among clinical isolates is far lower 90% (Li et al., 2015; Javed et al., 2016, 2018; World Health Organization, 2016; Maningi et al., 2018). According to the standard (World health organization, 2014) and the actual situations in clinical practice of new molecular DST assays (Li et al., 2015; Javed et al., 2016, 2018; World Health Organization, 2016; Maningi et al., 2018), the sensitivity for STR by WGS in the present study was recognized to achieve to an acceptable degree (using *rpsL* and *rrs* 530 loop and 912 loop, 83%), and the specificity was excellent (97.8%) while the sensitivity of *embB* for predicting EMB resistance was excellent (90.9%), but the specificity (65.1%) was far lower than the standard, which requires more than 95% (World health organization, 2014).

The results in the present study show that the sensitivity of drug resistance-associated genes or intergenic regions, whether alone or combined, could not predict 100% of the interested drug resistance, which is similar to that reported by most studies (Li et al., 2010; Farhat et al., 2016; Miotto et al., 2017; Shea et al., 2017). For the gap between genotypic and phenotypic resistance, one most fundamental cause may be that current sequencing technologies have varying capabilities to detect low frequencies (<20%) of resistant strains mixed with susceptible strains relative to phenotypic testing that can detect resistant strains making up only 1% of the total population (Canetti et al., 1969; Do and Dobrovic, 2009; Oh et al., 2010; Miotto et al., 2017). Second, breakpoint artifacts (i.e., inappropriately high critical concentrations) can be a major source of misclassification of phenotypes (Miotto et al., 2017), e.g., the threshold value for EMB resistance used in L-J medium mentioned in Zhang et al.’s (2009) report. Third, synonymous mutations are universally acknowledged to be unrelated with drug resistance as they do not cause any change in the structure of the protein (Torres et al., 2015), so synonymous mutations are excluded in the present study; however, drug-resistant strains are more likely to carry synonymous mutations although no statistical differences were found for most of loci (Supplementary Table 5), which cut down the sensitivity of sequencing. Previous studies show that these mutations can sometimes confer resistance (Safi et al., 2013; Van Deun et al., 2013). Fourth, predicting drug resistance based on WGS data relies on the knowledge of drug-resistance mechanisms; however, drug-resistance mechanisms have not been understood clearly, which results in WGS mispredicting resistance or susceptibility according to including mutations or without mutations, respectively. Previous studies (Li et al., 2010; Farhat et al., 2016; Miotto et al., 2017; Shea et al., 2017) as well as the present study report certain phenotypically susceptible isolates carried mutations while phenotypically resistant isolates show wild types in interested genes, especially in noncanonical genes associated with resistance. Applied proteomics and bioinformatics analysis (such as molecular docking, pupylation, and protein–protein interaction) on uncharacterized and hypothetical proteins in *M. tuberculosis* might give a clue for the novel mechanism of drug resistance (Sharma and Bisht, 2017a, b; Sharma et al., 2018).

## Conclusion

The data in this study raise our understanding of the molecular determinants of resistance to INH, RMP, EMB, and STR; high sensitivities and specificities of mutations in the genes of *katG* (INH), *rpoB* (RMP), *rpsL* (STR), and *rrs* 530 loop and 912 loop (STR) provide us a good choice to predict INH, RMP, and STR resistance by WGS or target region sequencing in the future. Further, the results provide clues in clarifying the drug-resistance mechanisms of *M. tuberculosis* isolates from China.

## Data Availability Statement

The datasets generated for this study can be found in the NCBI with accession numbers SRA065095 and PRJNA633954.

## Ethics Statement

This study was approved by the ethics committee of the National Institute for Communicable Disease Control and Prevention, Chinese Center for Disease Control and Prevention. The patients with TB were included in the present research only after we received informed written consent from themselves or from their parents/guardians in cases in which the patient was a child (18 years of age). An assent was also obtained from participants between 14 and 18 years of age.

## Author Contributions

GL designed and conducted this study. LW and GL wrote the first drafts of the manuscript. LW, HL, and ML sequenced the isolates and performed molecular characterization. YJ, XZ, and ZL performed drug susceptibility testing and spoligotyping. KW collected *M. tuberculosis* strains and provided oversight for sequencing and bioinformatics support. C-XG provided key manuscript edits. All authors commented on the manuscript draft and read and approved the final manuscript.

## Conflict of Interest

The authors declare that the research was conducted in the absence of any commercial or financial relationships that could be construed as a potential conflict of interest.

## Footnotes

1 http://www.pasteur-guadeloupe.fr:8081/SITVITDemo/index.jsp

## Supplementary Material

TABLE S1 Drug susceptibility patterns of 183 clinical *M. tuberculosis* for whole-genome sequencing.

TABLE S2 Synonymous mutations in 183 *M. tuberculosis* and their associations with drug resistance.

TABLE S3 Associations between synonymous mutations and Beijing genotype of 183 *M. tuberculosis* clinical isolates.

TABLE S4 Associations between mutations *katG*463 or *gidB*92 and drug resistance in 183 *M. Tuberculosis*.

TABLE S5 Associations between mutations *katG*463 or *gidB*92 and Beijing genotype in 183 *M. Tuberculosis*.

TABLE S6 Characterizations of *katG* mutations and its combination mutations with other 10 genes or regions associated with isoniazid resistance among 137 multi-drug resistant isolates.

TABLE S7 Mutation characterizations of *rpoB* among 137 MDR isolates from China.

TABLE S8 Mutation characterizations of *gidB* among 100 streptomycin resistant isolates and 83 streptomycin susceptible isolates from China.

TABLE S9 Mutation characterizations of *embABC* among 77 ethambutol resistant isolates and 106 ethambutol susceptible isolates from China.
