# Supplementary material for: Genomic Analysis Identifies Mutations Concerning Drug-Resistance and Beijing Genotype in Multidrug-Resistant Mycobacterium tuberculosis Isolated From China
Source: Front Microbiol. 2020 Jul 15;11:1444. doi: 10.3389/fmicb.2020.01444 (PMC7373740; doi:10.3389/fmicb.2020.01444)
Supplement: TABLE S1 — Drug susceptibility patterns of 183 clinical M. tuberculosis for whole-genome sequencing. [file Table_1.docx]

Supplemental Table 1 Drug susceptibility patterns of 183 clinical *M. tuberculosis* for whole-genome sequencing

| Susceptibility or resistance | Number of strains |
| --- | --- |
| Fully susceptible^*^ | 46 |
| MDR except XDR | 113 |
| HR | 10 |
| HRE | 12 |
| HRO | 3 |
| HRS | 31 |
| HREC | 2 |
| HREO | 6 |
| HRSE | 20 |
| HRSO | 10 |
| HRSCK | 1 |
| HRSEC | 1 |
| HRSEO | 17 |
| Over all XDR | 24 |
| HRKO | 1 |
| HRSCO | 2 |
| HRSKO | 1 |
| HRSECO | 1 |
| HRSEKO | 5 |
| HRECKO | 3 |
| HRSCKO | 1 |
| HRSECKO | 10 |

Note, * isolates susceptible to isoniazid, rifampicin, ethambutol, streptomycin, kanamycin, ofloxacin, and capreomycin, simultaneously; H, isoniazid; R, rifampicin; S, streptomycin; E, ethambutol; K, kanamycin; O, ofloxacin; C, capreomycin.
