# Supplementary material for: Genomic Analysis Identifies Mutations Concerning Drug-Resistance and Beijing Genotype in Multidrug-Resistant Mycobacterium tuberculosis Isolated From China
Source: Front Microbiol. 2020 Jul 15;11:1444. doi: 10.3389/fmicb.2020.01444 (PMC7373740; doi:10.3389/fmicb.2020.01444)
Supplement: TABLE S3 — Associations between synonymous mutations and Beijing genotype of 183 M. tuberculosis clinical isolates. [file Table_3.docx]

Supplemental Table 3 Associations between synonymous mutations and Beijing genotype of 183 *M. tuberculosis* clinical isolates

| Drug | Gene | Mutations | Occuring rate in Beijing genotype isolates | Occuring rate in non-Beijing genotype isolates | *χ^2^* | *P* |
| --- | --- | --- | --- | --- | --- | --- |
| INH | *katG* | 428 GGG-GGA (Gly-Gly) | 1/141 | 0/42 | — | — |
|  |  | 40 CCC-CCT (Pro-Pro) | 2/141 | 0/42 | — | — |
|  | *ahpC* | 125 CTG-CTA（Leu-Leu） | 1/141 | 0/42 | — | — |
|  |  | 165 CTG-TTG（Leu-Leu） | 1/141 | 0/42 | — | — |
|  | *ndh* | 193 GCC-GCA (Ala-Ala) | 2/141 | 0/42 | — | — |
|  |  | 226 GCA-GCC (Ala-Ala) | 0/141 | 2/42 | — | — |
|  |  | 83 CAG-CAA (Gln-Gln) | 1/141 | 0/42 | — | — |
|  | *efpA* | 114 ACC-ACT (T-T) | 1/141 | 0/42 | — | — |
|  |  | 318 GGC-GGT (G-G) | 1/141 | 0/42 | — | — |
|  |  | 83CTG-TTG (L-L) | 0/141 | 2/42 | — | — |
|  | *iniA* | 178 GGT-GGC (G-G) | 1/141 | 15/42 | 45.41 | 0.000 |
|  |  | 126 CCC-CCA (P-P) | 0/141 | 2/42 | — | — |
|  |  | 320 TTG-TTA (L-L) | 0/141 | 1/42 | — | — |
|  |  | 501 TCG-TCT (S-S) | 1/141 | 0/42 | — | — |
|  | *iniB* | 220 GTC-GTT (V-V) | 1/141 | 0/42 | — | — |
|  |  | 395 GGC-GGG (G-G) | 1/141 | 0/42 | — | — |
|  |  | 398 GGC-GGA (G-G) | 1/141 | 0/42 | — | — |
|  |  | 398 GGC-GGT (G-G) | 1/141 | 0/42 | — | — |
|  |  | 402 CCA-CCC (P-P) | 0/141 | 1/42 | — | — |
|  |  | 420 ACG-ACC (T-T) | 1/141 | 0/42 | — | — |
|  | *iniC* | 16 CAG-CAA (Q-Q) | 0/141 | 1/42 | — | — |
|  |  | 22 CCG-CCA (P-P) | 1/141 | 0/42 | — | — |
|  |  | 147 ACG-ACA (T-T) | 1/141 | 0/42 | — | — |
| RMP | *rpoB* | 1075 GCT-GCC (Ala-Ala) | 139/141 | 7/42 | 134.6 | 0.000 |
|  |  | 1063 GGG-GGC (Gly-Gly) | 2/141 | 0/42 | — | — |
|  |  | 782 GGC-GGT (Gly-Gly) | 0/141 | 1/42 | — | — |
|  |  | 876 GGT-GGG (Gly-Gly) | 0/141 | 2/42 | — | — |
| EMB | *embA* | 76 TGC-TGT (C-C） | 136/141 | 5/42 | 130.8 | 0.001 |
|  |  | 1092 GCG-GCA (A-A) | 2/141 | 13/42 | 33.69 | 0.000 |
|  |  | 253 GCT-GCG (A-A) | 0/141 | 1/42 | — | — |
|  |  | 580 CTG-CTA (L-L) | 0/141 | 1/42 | — | — |
|  |  | 633 CCG-CCC (P-P) | 0/141 | 1/42 | — | — |
|  |  | 347 CTG-TTG (L-L) | 1/141 | 0/42 | — | — |
|  |  | 413 ATC-ATT (I-I) | 1/141 | 0/42 | — | — |
|  |  | 454 GCG-GCA (A-A) | 1/141 | 1/42 | — | — |
|  |  | 644 CAC-CAT (H-H) | 1/141 | 0/42 | — | — |
|  |  | 803 AAC-AAT (N-N) | 1/141 | 0/42 | — | — |
|  | *embB* | 1033 AAC-AAT (N-N) | 1/141 | 0/42 | — | — |
|  |  | 304 CTG-TTG (L-L) | 1/141 | 0/42 | — | — |
|  |  | 517 GCG-GCC(A-A) | 0/141 | 1/42 | — | — |
|  |  | 534 GAC-GAT (D-D) | 8/141 | 0/42 | — | — |
|  |  | 1023 CTG-CTA (L-L) | 0/141 | 1/42 | — | — |
|  |  | 766 CCG-CCT (P-P) | 0/141 | 1/42 | — | — |
|  | *embC* | 927 CGC-CGT (R-R) | 139/139 | 41/41 | — | — |
|  |  | 213 AGC-AGT (S-S) | 1/139 | 0/41 | — | — |
|  |  | 227 ACG-ACA (T-T) | 1/139 | 0/41 | — | — |
|  |  | 264 CCC-CCG (P-P) | 0/139 | 2/41 | — | — |
|  |  | 789 GGC-GGA (G-G) | 1/139 | 0/41 | — | — |
|  |  | 968 GTG-GTA (V-V) | 1/139 | 0/41 | — | — |
| STR | *rpsL* | 13 CGG-CGA (Arg-Arg) | 0/141 | 1/42 | — | — |
|  |  | 113 GCA-GCG (Ala-Ala） | 0/141 | 1/42 | — | — |
|  | *gidB* | 19 GCT-GCC (Ala-Ala) | 1/140 | 0/42 | — | — |
|  |  | 216 GCA-GCC (Ala-Ala) | 1/140 | 0/42 | — | — |
|  |  | 7 GCG-GCA (Ala-Ala) | 3/140 | 0/42 | — | — |
|  |  | 205 GCA-GCG (Ala-Ala) | 138/140 | 7/42 | 133.8 | 0.000 |

Note, INH, isoniazid; RMP, rifampicin; EMB, ethambutol; STR, streptomycin; “—” meaned the values were not acquired because of the small samples.
