# Supplementary material for: Genomic Analysis Identifies Mutations Concerning Drug-Resistance and Beijing Genotype in Multidrug-Resistant Mycobacterium tuberculosis Isolated From China
Source: Front Microbiol. 2020 Jul 15;11:1444. doi: 10.3389/fmicb.2020.01444 (PMC7373740; doi:10.3389/fmicb.2020.01444)
Supplement: TABLE S4 — Associations between mutations katG463 or gidB92 and drug resistance in 183 M. Tuberculosis. [file Table_4.docx]

Supplemental Table 4 Associations between mutations *katG*463 or *gidB*92 and drug resistance in 183 *M. tuberculosis*

| Drug | Gene | Mutations | Occuring rate in resistant isolates | Occuring rate in susceptible isolates | *χ^2^* | *P* |
| --- | --- | --- | --- | --- | --- | --- |
| INH | *katG* | 463 CGG-CTG (Arg-Leu) | 115/137 | 31/46 | 5.85 | 0.016 |
| STR | *gidB* | 92 GAA-GAC (Glu-Asp) | 87/99 | 56/83 | 11.17 | 0.001 |

Note, INH, isoniazid; STR, streptomycin.
