# Supplementary material for: Genomic Analysis Identifies Mutations Concerning Drug-Resistance and Beijing Genotype in Multidrug-Resistant Mycobacterium tuberculosis Isolated From China
Source: Front Microbiol. 2020 Jul 15;11:1444. doi: 10.3389/fmicb.2020.01444 (PMC7373740; doi:10.3389/fmicb.2020.01444)
Supplement: TABLE S5 — Associations between mutations katG463 or gidB92 and Beijing genotype in 183 M. Tuberculosis. [file Table_5.docx]

Supplemental Table 5 Associations between mutations *katG*463 or *gidB*92 and Beijing genotype in 183 *M. tuberculosis*

| Drug | Gene | Mutations | Occuring rate in Beijing genotype isolates | Occuring rate in non-Beijing genotype isolates | *χ^2^* | *P* |
| --- | --- | --- | --- | --- | --- | --- |
| INH | *katG* | 463 CGG-CTG (Arg-Leu) | 139/141 | 7/42 | 134.6 | 0.000 |
| STR | *gidB* | 92 GAA-GAC (Glu-Asp) | 138/140 | 5/42 | 144.1 | 0.000 |

Note, INH, isoniazid; STR, streptomycin.
