# Supplementary material for: Genomic Analysis Identifies Mutations Concerning Drug-Resistance and Beijing Genotype in Multidrug-Resistant Mycobacterium tuberculosis Isolated From China
Source: Front Microbiol. 2020 Jul 15;11:1444. doi: 10.3389/fmicb.2020.01444 (PMC7373740; doi:10.3389/fmicb.2020.01444)
Supplement: TABLE S6 — Characterizations of katG mutations and its combination mutations with other 10 genes or regions associated with isoniazid resistance among 137 multi-drug resistant isolates. [file Table_6.docx]

Supplemental Table 6 Characterizations of *katG* mutations and its combination mutations with other 10 genes or regions associated with isoniazid resistance among 137 multi-drug resistant isolates

| *katG* | No. of isolates | Combined mutations in *inhA* promoter or *oxyR-ahpC* intergenic region | Combined mutations in *inhA*, *ahpC*, *ndh*, *kasA* or *efpA* | Combined mutations in *iniA*, *iniB* or *iniC* |
| --- | --- | --- | --- | --- |
| No mutation | 13 | 3 *inhA* (-15)C-T; 10 WT | 1 *ndh* 195CAC-AAC(His-Asn); 1 *efpA* 275CTG-ATG(L-M); 11 WT | 1 *iniB* 171GGC-GAC(G-D); 1 *iniB* 399ATG-AAC(M-N) and 400AAC-ACC(N-T); 1 *iniB* nucletide positions 653-664 CTGGTGTCGGCG deleted; 10 WT |
| 90TGG-AGG (Trp-Arg) | 1 | 1 *ahpC* (-48)G-A | 1 WT | 1 WT |
| 109GCT-ACT (Ala-Thr) | 1 | 1 *inhA* (-15)C-T | 1 WT | 1 WT |
| 120GGC-AGC(Gly-Ser), 408TTC-CTC (Phe-Leu) | 1 | 1 WT | 1 *ndh* nucleotide positions 554-568 deleted TGGCCGGACAGATCG and 575-583 deleted TGGCCGAGC | 1 WT |
| 138(AAC-GAC)(Asn-Asp) | 1 | 1 *inhA* (-8)T-A | 1 WT | 1 WT |
| 142(GAC-GGC)(Asp-Gly) | 2 | 2 WT | 2 WT | 2 WT |
| 155(TAC-TGC)(Tyr-Cys), 587（CTG-CGG)(Leu-Arg) | 1 | 1 *ahpC* (-72) C-T | 1 WT | 1 WT |
| 163(GAC-AAC)(Asp-Asn) | 1 | 1 *ahpC* (-54)C-T, | 1 WT | 1 *iniB* 400AAC-ACC(N-T) |
| 169(GGC-AGC)(Gly-Ser) | 4 | 3 *inhA* (-15)C-T, 1 *inhA* (-15)C-T and *ahpC* (-72)C-T | 1 *ndh* nucleotide positions 560-578 deleted GACAGATCGCCGAGCTGGCCG; 3 WT | 4WT |
| 189(GAC-GCC)(Asp-Ala) | 1 | 1 WT | 1 *ndh* 197TTG-GTG(Leu-Val) | 1 WT |
| 189(GAC-GGC)(Asp-Gly) | 1 | 1 WT | 1 WT | 1 WT |
| 191（TGG-CGG)(Trp-Arg) | 5 | 4 *inhA* (-15)C-T, 1 *inhA* (-15)C-T and *ahpC* (-48) G-A | 1 *inhA* 194ATC-ACC（Ile-Thr); 1 *ndh* 195 CAC-CCC(His-Pro); 3 WT | 5 WT |
| 232(CCG-TCG)(Pro-Ser),419(GAC-GGC)(Asp-Gly) | 1 | 1 WT | 1 *ndh* 195CAC-CAA(His-Gln) | 1 *iniB* 403GTG-ATG(V-M) |
| 234(GGG-GAG)(Gly-Glu) | 1 | 1 WT | 1 WT | 1 WT |
| 235(CCG-TCG)(Pro-Ser), 302(AGC-CGC)(Ser-Arg) | 1 | 1 WT | 1 WT | 1 WT |
| 299(GGC-AGC)(Gly-Ser) | 1 | 1 WT | 1 WT | 1 WT |
| 315(AGC-AAC)(Ser-Asn),379(GCC-ACC)(Ala-Thr) | 1 | 1 WT | 1 WT | 1 WT |
| 315(AGC-AAC)(Ser-Asn) | 10 | 10 WT | 1 *ndh* 196 ACG-GCG (Thr-Ala); 1 *ndh* 194 GAG-TAG (Glu-Ter); 8 WT | 1 *iniB* 396 TAT-TAG(Y-*) and 400 AAC-ACC(N-T); 1 *iniB* 400 AAC-ACC(N-T); 1 *iniB* nucletide positions 648-659 TGGCGCTGGTGT deleted; 7 WT |
| 315(AGC-ACA)(Ser-Thr) | 1 | 1 WT | 1 WT | 1 *iniB* nucletide positions 1171-1196 GGCGGTACCGGCGGCTATGGCGGCAT deleted and 400AAC-ACC(N-T) |
| 315(AGC-ACC)(Ser-Thr) | 78 | 2 *inhA* (-8)T-G; 2 *inhA* (-8)T-C; 1 *inhA* (-8)T-A; 1 *inhA* (-15)C-T; 1 *inhA* (-34)C-T; 1 *ahpC* (-52)C-T; 70 WT | 1 *ndh* 154GCT-GTT(Ala-Val); 1 *ahpC* 148 AGC-AGA(Ser-Arg）; 1 *kasA* 142GTT-ATT(V-I); 1 *inhA* 94(TCG-GCG)(Ser-Ala) and *ndh* nucletide positions 563-579 deleted AGATCGCCGAGCTGGCC; 1 *kasA* 253CAC-TAC(H-Y); 1 *ndh* 194GAG-CAG(Glu-Gln) and *kasA* 253CAC-TAC(H-Y); 1 *efpA* 313ATC-GTC(I-V); 1 *ndh* 65GAG-GGG(Glu-Gly); 1 *efpA* 7ACA-CCA(T-P) and 227GCC-GAC(A-D); 1 *inhA* 95(ATT-CTT)(Ile-Leu); 1 *ndh*  196ACG-CCG(Thr-Pro); 1 *ndh*  195CAC-CCC(His-Pro); 1 *ndh*  nucletide positions 591-609 deleted GAAGGGCGCATTCCGGCAC; 1 *ndh* 197TTG-GTG(Leu-Val); 64 WT | 1 *iniB* 357CTG-CGG(L-R); 1 *iniB* 395GGC-TGC(G-C); 1 *iniB* 396TAT-TAG(Y-*); 1 *iniB* 397GGC-TGC(G-C）; 1 *iniB* 399ATG-ATA(M-I) and 400AAC-ACC(N-T); 6 *iniB* 400AAC-ACC(N-T); 1 *iniB* 415GCC-CCC(A-P); 1 *iniC* 93ACC-ATC (T-I); 4 *iniC* nucletide position 174 C deleted; 1 *iniB* nucletide positions 1175-1177 inserted GCG, 393ACC-CCC(T-P) and 400AAC-ACC(N-T); 1 *iniB* nucletide positions 646-657 deleted GTTGGCGCTGGT; 2 *iniB* nucletide positions 648-659 deleted TGGCGCTGGTGT and 400AAC-ACC(N-T); 2 *iniB* nucletide positions 648-659 TGGCGCTGGTGT deleted; 1 *iniA* 306GGT-CCT(R-P); 1 *iniA* 323TCT-TTT(S-F); 2 *iniA* nucletide posistion 522 inserted A; 1 *iniB* 307TTG-GTG(L-V), 400AAC-ACC(N-T) and *iniC* nucletide position 174 C deleted; 50WT |
| 315(AGC-ACC)(Ser-Thr)，649(GCC-ACC)(Ala-Thr) | 1 | 1 WT | 1 WT | 1 WT |
| 315(AGC-GGC)(Ser-Gly) | 1 | 1 *ahpC* (-52)C-A | 1 WT | 1 *iniB* 398GGC-TGC(G-C), 399ATG-ATA(M-I）and 400AAC-ACC(N-T) |
| 378(CTG-CCG)(Leu-Pro) | 1 | 1 *ahpC* (-72)C-T | 1 WT | 1 *iniB* nucletide positions 653-664 CTGGTGTCGGCG deleted |
| 380(ACT-ATT)(Thr-Ile) | 1 | 1 *inhA* (-15)C-T and (-54)C-T | 1 WT | 1 WT |
| 380(ACT-CCT)(Thr-Pro) | 1 | 1 *inhA* (-15)C-T | 1 WT | 1 *iniA* nucletide posistion 842 A deleted |
| 419(GAC-CAC)(Asp-His) | 1 | 1 *inhA* (-47) insert T | 1 WT | 1 WT |
| nucleotide positions 861-866 deleted ACCCGA | 1 | 1 WT | 1 WT | 1 *iniB* 400AAC-ACC(N-T) and *iniC* 228GAC-GAA(D-E) |
| nucleotide positions 86-88 deleted CCG and 155(TAC-TGC)(Tyr-Cys) | 1 | 1 WT | 1 WT | 1 WT |
| nucleotide position 956 deleted T | 1 | 1 *ahpC* (-81)C-T | 1 *ndh* 89GTC-TTC(Val-Phe) | 1 WT |
| nucleotide positions 1-2 deleted GT, 5(CAC-CCC)(His-Pro), 12(ACC-CCC)(Thr-Pro), 13(ACC-CCC)(Thr-Pro), 20(TGT-TGC)(Cys-Cys) | 1 | 1 WT | 1 WT | 1 *iniB* 399ATG-CTG(M-L) and 403GTG-GGG(V-G) |
