# Supplementary material for: Genomic Analysis Identifies Mutations Concerning Drug-Resistance and Beijing Genotype in Multidrug-Resistant Mycobacterium tuberculosis Isolated From China
Source: Front Microbiol. 2020 Jul 15;11:1444. doi: 10.3389/fmicb.2020.01444 (PMC7373740; doi:10.3389/fmicb.2020.01444)
Supplement: TABLE S7 — Mutation characterizations of rpoB among 137 MDR isolates from China. [file Table_7.docx]

Supplemental Table 7 Mutation characterizations of *rpoB* among 137 MDR isolates from China

| Mutations^*^ | Frequency (*No.* of isolates) | Relative frequency^a^ (%) |
| --- | --- | --- |
| No mutation | 8 | 5.8% |
| 170GTC-TTC(Val-Phe), 920ATG-GTG(Met-Val) | 1 | 0.1 |
| 430CTG-CCG(Leu-Pro), 427ACC-CCC(Thr-Pro) | 1 | 0.1 |
| 430CTG-CCG(Leu-Pro), 424TTC-CTC(Phe-Leu) | 1 | 0.1 |
| 430CTG-CCG(Leu-Pro), 431AGC-GGC(Ser-Gly) | 1 | 0.1 |
| 435GAC-GGC(Asp-Gly), 430CTG-CGG(Leu-Arg) | 1 | 0.1 |
| 435GAC-GCC(Asp-Ala), 452CTG-CCG(Leu-Pro) | 1 | 0.1 |
| 435GAC-GGC(Asp-Gly) | 1 | 0.1 |
| 435GAC-GGC(Asp-Gly), 430CTG-CCG(Leu-Pro) | 1 | 0.1 |
| 435GAC-GGC(Asp-Gly), 452CTG-CCG(Leu-Pro) | 3 | 2.2 |
| 435GAC-GTC(Asp-Val) | 4 | 2.9 |
| 435GAC-TAC(Aap-Tyr) | 1 | 0.1 |
| 435GAC-TAC(Aap-Tyr)，441TCG-ACG(Ser-Thr) | 1 | 0.1 |
| 435GAC-TAC(Asp-Tyr), 172CAG-CGG(Gln-Arg) | 1 | 0.1 |
| 441TCG-ATG(Ser-Met) | 1 | 0.1 |
| 441TCG-TTG(Ser-Leu), 469GTG-TTG(Val-Leu) | 1 | 0.1 |
| 445CAC-AAC(His-Asn), 170GTC-TTC(Val-Phe), nucleotide positions 3016-3019 inserted TGCG,925GAC-GGG(Asp-Gly), 1009AGC-GGG(Ser-Gly) | 1 | 0.1 |
| 445CAC-AAC(His-Asn), 430CTG-CCG(Leu-Pro) | 1 | 0.1 |
| 445CAC-AAC(His-Asn), 454CCC-CTC(Pro-Leu) | 1 | 0.1 |
| 445CAC-CGC(His-Arg) | 1 | 0.1 |
| 445CAC-CGC(His-Arg), 675GGC-GAC(Gly-Asp) | 1 | 0.1 |
| 445CAC-CTC(His-Leu) | 1 | 0.1 |
| 445CAC-CTC(His-Leu), 428AGC-GGC(Ser-Gly) | 3 | 2.2 |
| 445CAC-GAC(His-Asp) | 7 | 5.1 |
| 445CAC-GAC(His-Asp), 435GAC-GGC(Asp-Gly) | 1 | 0.1 |
| 445CAC-GAC(His-Asp), 1056CAG-CAC(Gln-His) | 1 | 0.1 |
| 445CAC-GAC(His-Asp), nucleotide positions 3011-3017 deleted TCTTCGA | 1 | 0.1 |
| 445CAC-GAC(His-Asp), 435GAC-TAC(Aap-Tyr) | 1 | 0.1 |
| 445CAC-TAC(His-Tyr) | 4 | 2.9 |
| 445CAC-TAC(His-Tyr), 280CCC-CTC(Pro-Leu) | 1 | 0.1 |
| 445CAC-TAC(His-Tyr), 378CTG-CGG(Leu-Arg) | 1 | 0.1 |
| 445CAC-TAC(His-Tyr), 391GAG-GGG(Glu-Gly) | 1 | 0.1 |
| 445CAC-TAC(His-Tyr), 400ACC-ATC(Thr-Ile) | 1 | 0.1 |
| 445CAC-TAC(His-Tyr), 429CAG-CAC(Gln-His) | 1 | 0.1 |
| 445CAC-TAC(His-Tyr), 460GAG-GGG(Glu-Gly) | 1 | 0.1 |
| 445CAC-TAC(His-Tyr), 672TCC-TAC(Ser-Tyr) | 1 | 0.1 |
| 445CAC-TTC(His-Phe), 226CAA-CGA(Gln-Arg) | 1 | 0.1 |
| 450TCG-TTG(Ser-Leu) | 56 | 40.9 |
| 450TCG-TTG(Ser-Leu), 45CCG-CGG(Pro-Arg) | 1 | 0.1 |
| 450TCG-TTG(Ser-Leu), 45CCG-TCG(Pro-Ser) | 2 | 2.2 |
| 450TCG-TTG(Ser-Leu), 376GGC-GTC(Gly-Val) | 1 | 0.1 |
| 450TCG-TTG(Ser-Leu), nucleotide positions 1651-1653 deleted AGC | 1 | 0.1 |
| 450TCG-TTG(Ser-Leu), nucleotide positions 3028-3039 inserted CGGGGCGCGGGG | 1 | 0.1 |
| 450TCG-TTG(Ser-Leu), 454CCC-CTC(Pro-Leu), 593CAC-TAC(His-Tyr) | 1 | 0.1 |
| 450TCG-TTG(Ser-Leu), 480ATC-ACC(Ile-Thr)，1080CAG-CGG(Gln-Arg) | 1 | 0.1 |
| 450TCG-TTG(Ser-Leu), 534GTG-ATG(Val-Met) | 1 | 0.1 |
| 450TCG-TTG(Ser-Leu), 534GTG-GCG(Val-Ala) | 1 | 0.1 |
| 450TCG-TTG(Ser-Leu), 545GAC-GAG(Asp-Glu) | 1 | 0.1 |
| 450TCG-TTG(Ser-Leu), 554CTG-CCG(Leu-Pro) | 1 | 0.1 |
| 450TCG-TTG(Ser-Leu), 675GGC-GAC(Gly-Asp) | 1 | 0.1 |
| 450TCG-TTG(Ser-Leu), 834CCG-ACG(Pro-Thr) | 1 | 0.1 |
| 452CTG-CCG(Leu-Pro) | 3 | 2.2 |
| 452CTG-CCG(Leu-Pro), 481GAA-GCA(Glu-Ala) | 1 | 0.1 |
| 675GGC-GAC(Gly-Glu) | 1 | 0.1 |
| nucleotide positions 1296-1304 deleted ATTCATGGA | 1 | 0.1 |
| nucleotide positions 1310-1312 deleted ACA, 491ATC-ACC(Ile-Thr) | 1 | 0.1 |

Note, *the codon number were changed according to the sequence of H37RV（accession *No.* : NC_000962.2）, ^a^ Compared with the total number of isolates resistant to rifampicin
