# Supplementary material for: Genomic Analysis Identifies Mutations Concerning Drug-Resistance and Beijing Genotype in Multidrug-Resistant Mycobacterium tuberculosis Isolated From China
Source: Front Microbiol. 2020 Jul 15;11:1444. doi: 10.3389/fmicb.2020.01444 (PMC7373740; doi:10.3389/fmicb.2020.01444)
Supplement: TABLE S8 — Mutation characterizations of gidB among 100 streptomycin resistant isolates and 83 streptomycin susceptible isolates from China. [file Table_8.docx]

Supplemental Table 8 Mutation characterizations of *gidB* among 100 streptomycin resistant isolates and 83 streptomycin susceptible isolates from China

| Group (No. of isolates) | Mutations in *gidB* | No. of isolates | Conmbined mutations in *rpsL* and *rrs* 530 loop and 912 loop |
| --- | --- | --- | --- |
| Streptomycin resistant isolates (100) | 28 GGA-GAA(Gly-Glu) | 1 | *rrs* 518C-T |
|  | 32 GAG-GAC(Glu-Asp) | 1 | *rpsL* 88AAG-AGG(Lys-Arg) |
|  | 60 GAA-TAA(Glu-Ter) | 1 | WT |
|  | 79 TTG-TTT(Leu-Phe) | 1 | WT |
|  | nucletide position 102 deleted G | 1 | WT |
|  | nucletide position 105 deleted C | 1 | *rpsL* 43AAG-AGG(Lys-Arg) |
|  | 115 GTG-GGG(Val-Gly) | 1 | WT |
|  | 146 ACG-AAG(Thr-Lys) | 1 | *rrs* 888G-T |
|  | 154 CGG-TGG(Arg-Trp) | 1 | WT |
|  | 163 AAA-TAA(Lys-Ter) | 2 | T |
|  | 202 GTG-GCG(Val-Ala) | 1 | WT |
|  | WT | 88 | －^*^ |
| Streptomycin susceptible isolates (83) | 167GCT-CCT(Ala-Pro) | 1 | WT |
|  | 223ACG-ATG(Thr-Met) | 1 | WT |
|  | 30GGT-GTT(Gly-Val), 87CAG-TAG(Gln-Ter) | 1 | WT |
|  | 86CTC-TTC(Leu-Phe) | 1 | WT |
|  | nucletide position 102 deleted G | 3 | WT |
|  | WT | 74 | 2 *rpsL* 43AAG-AGG(Lys-Arg); 1 *rpsL* 88AAG-AGG(Lys-Arg); 1 *rrs* nucletide positions 334-344 deleted CAGACTCCTAC; 1 *rrs* nucletide positions 388-394 deleted ATGCAGC; 1 *rrs* nucletide positions 390-394 deleted GCAGC; 2 *rrs* nucletide positions 392-394 deleted AGC; 1 *rrs* 555A-T; 2 *rrs* 846C-T and 1017G-C; 65 WT |

Note, ^*^The combined mutations were not listed here for that 79 out 88 isolates with wild type of *gidB* carried mutations in *rpsL* and *rrs* 530 loop and 912 loop; WT, wild type.
