# Supplementary material for: Genomic Analysis Identifies Mutations Concerning Drug-Resistance and Beijing Genotype in Multidrug-Resistant Mycobacterium tuberculosis Isolated From China
Source: Front Microbiol. 2020 Jul 15;11:1444. doi: 10.3389/fmicb.2020.01444 (PMC7373740; doi:10.3389/fmicb.2020.01444)
Supplement: TABLE S9 — Mutation characterizations of embABC among 77 ethambutol resistant isolates and 106 ethambutol susceptible isolates from China. [file Table_9.docx]

Supplemental Table 9 Mutation characterizations of *embABC* among 77 ethambutol resistant isolates and 106 ethambutol susceptible isolates from China

| Group | Genes | Mutations | No.(%^*^) of isolates) | Other mutations |
| --- | --- | --- | --- | --- |
| Ethambutol resistant isolates (77) | *embA* | 200GGC-AGC(G-S) | 1 (1.3) | 1 *embB* D354N, A689T and *embC* P150S |
|  |  | 331GCC-ACC(A-T) | 1 (1.3) | 1 *embB* M306V |
|  |  | 366GCG-GAG(A-E) | 2 (2.6) | 2 *embB* A505T |
|  |  | 4GAC-AAC(D-N) | 1 (1.3) | 1 *embB* M306V |
|  |  | 769CCC-ACC(P-T) | 1 (1.3) | 1 *embB* M306V |
|  |  | 867CCG-CTG(P-L) | 1 (1.3) | 1 *embB* M306V |
|  |  | No mutation | 70 (90.9) |  |
|  | *embB* | 73GGG（G-R) | 1 (1.3) |  |
|  |  | 306ATG-ATA(M-I),406GGC-AGC(G-S) | 1 (1.3) |  |
|  |  | 306ATG-ATC(M-I),643ACC-ATC(T-I) | 1 (1.3) | ` |
|  |  | 306ATG-ATA(M-I) | 14 (18.2) |  |
|  |  | 306ATG-CTG(M-L) | 2 (2.6) |  |
|  |  | 306ATG-GTA(M-V) | 27 (35.1) | 1 *embA* P769T, 1 *embC* Q725R, 1 *embA* A331T and *embC* P150S, 1 *embA* D4N, 1 *embA* P867L, 1 *embC* P872A and T873P,and N874H |
|  |  | 319TAT-GAT(Y-D) | 1 (1.3) |  |
|  |  | 319TAT-TGT(Y-C) | 2 (2.6) |  |
|  |  | 354GAC-AAC(D-N),679GCC-ACC(A-T) | 1 (1.3) | 1 *embA* G200S |
|  |  | 354GAC-GCC(D-A) | 1 (1.3) | 1 *embC* V974L |
|  |  | 399AAC-ACC(N-T) | 1 (1.3) |  |
|  |  | 406GGC-AGC(G-S) | 3 (3.9) |  |
|  |  | 406GGC-GAC(G-D) | 2 (2.6) |  |
|  |  | 406GGC-GCC(G-A) | 4 (5.2) |  |
|  |  | 412TCG-CCG(S-P) | 1 (1.3) |  |
|  |  | 497CAG-CGG(Q-R) | 1 (1.3) |  |
|  |  | 505GCC-ACC(A-T) | 2 (2.6) | 2 *embA* A366E |
|  |  | 50GTG-GCG(V-A), 497CAG-AAG(Q-K) | 1 (1.3) |  |
|  |  | 50GTG-GCG(V-A),497CAG-CCG(Q-P) | 1 (1.3) |  |
|  |  | 642ACC-GCC(T-A), 1002 CAC-CGC (H-R) | 1 (1.3) |  |
|  |  | 1002CAC-CGC(H-R) | 1 (1.3) | ` |
|  |  | 1024GAC-AAC(D-N) | 1 (1.3) |  |
|  |  | No mutation | 7 (9.1) |  |
|  | *embC* | 150 CCG-TCG(P-S) | 1 (1.3) | 1 *embA* A331T and *embC* P150S |
|  |  | 725 CAG-CGG(Q-R) | 1 (1.3) | 1 *embB* M306V |
|  |  | 872 CCC-GCC(P-A), 873 ACC-CCC(T-P), 874 AAC-CAC(N-H) | 1 (1.3) | 1 *embB* M306V |
|  |  | 974 GTG-TTG(V-L) | 1 (1.3) | 1 *embB* D354A |
|  |  | No mutation | 73 (94.8) |  |
| Ethambutol susceptible isolates (106) | *embA* | 154GGT-AGT(G-S) | 1 (0.9) |  |
|  |  | 270ACC-CCC(T-P) | 1 (0.9) |  |
|  |  | 291GCC-ACC(A-T) | 1 (0.9) |  |
|  |  | 652ACG-AAG(T-K) | 2 (1.9) |  |
|  |  | 763CCG-TCG(P-S) | 1 (0.9) |  |
|  |  | 779GAG-CAG(E-Q) | 2 (1.9) |  |
|  |  | 951GAG-GAT(E-D) | 1 (0.9) | 1 *embB* G406D |
|  |  | No mutation | 97 (91.5) |  |
|  | *embB* | 84CCG-GGG(P-G),85 TGC-AGC(C-S), 86 GAC-GGG(D-G), 87 GTG-GCG(V-A), 88 GTG-GGG(V-G), 89 CGC-GGC(R-G), 563 ATT-CTT(I-L) | 1 (0.9) |  |
|  |  | 149 GAG-GCG(E-A) | 1 (0.9) |  |
|  |  | 186 GTC-GCC(V-A) | 1 (0.9) |  |
|  |  | 246 GGC-CGC(G-R) | 1 (0.9) | 1 *embC* A41S |
|  |  | 300 GAC-GGC(D-G) | 1 (0.9) |  |
|  |  | 306 ATG-ATA(M-I) | 4 (3.8) |  |
|  |  | 306 ATG-ATC(M-I) | 1 (0.9) |  |
|  |  | 306 ATG-CTG(M-L) | 4 (3.8) |  |
|  |  | 306ATG-CTG(M-L),563ATT-CTT(I-L) | 1 (0.9) |  |
|  |  | 306ATG-GTG(M-V) | 1 (0.9) | 1 *embC* V885M |
|  |  | 306ATG-GTG(M-V) | 3 (2.8) |  |
|  |  | 306ATG-GTG(M-V), 563ATT-CTT(I-L) | 1 (0.9) |  |
|  |  | 306ATG-GTG(M-V),497CAG-CAT(Q-H) | 1 (0.9) |  |
|  |  | 354GAC-AAC(D-N) | 1 (0.9) |  |
|  |  | 354GAC-GCC(D-A) | 1 (0.9) |  |
|  |  | 406 GGC-GAC(G-D) | 3 (2.8) |  |
|  |  | 406GGC-GCC(G-A) | 2 (1.9) |  |
|  |  | 497CAG-CGG(Q-R) | 1 (0.9) | 1 *embC* V42A |
|  |  | 497CAG-CGG(Q-R) | 1 (0.9) |  |
|  |  | 497CAG-AAG(Q-K) | 2 (1.9) |  |
|  |  | 563ATT-CTT(I-L) | 1 (0.9) |  |
|  |  | 603GGG-AGG(G-R) | 1 (0.9) | 1 *embC* V987G, L989G, nucletide positions 2969-2986 CATTCCCCTGCCAACGCC deleted, nucletide position 2987 C changed to G |
|  |  | 603GGG-AGG(G-R) | 1 (0.9) |  |
|  |  | 1024GAC-AAC(D-N) | 1 (0.9) |  |
|  |  | 1083 GCG-ACG(A-T) | 1 (0.9) |  |
|  |  | No mutation | 69 (65.1) |  |
|  | *embC* | 41GCT-TCT(A-S) | 1 (0.9) | 1 *embB* 246GGC-CGC(G-R) |
|  |  | 42 GTG-GCG(V-A) | 1 (0.9) | 1 embB 497CAG-CGG(Q-R) |
|  |  | 417 GTG-ATG(V-M | 2 (1.9) | 2 *embA* T652K |
|  |  | 557 ATT-ACT(I-T | 1 (0.9) |  |
|  |  | 738 CGG-CAG(R-Q) | 2 (1.9) |  |
|  |  | 885 GTG-ATG(V-M) | 1 (0.9) | 1 *embB* M306V |
|  |  | 927CGC-CGT(R-R), 987GTG-GGG(V-G), 989 CTG-GGG(L-G), nucletide positions 2969-2986 CATTCCCCTGCCAACGCC deleted, nucletide position 2987 C | 1 (0.9) | 1 *embB* G603R |
|  |  | No mutation | 97 (91.5) |  |

Note, ^*^For the ethambutol resistant *M. tuberculosis* isolates, the value in the bracket= the number of mutated or no mutated isolates/the number of ethambutol resistant *M. tuberculosis* isolates；for the ethambutol susceptible *M. tuberculosis* isolates, the value in the bracket= the number of mutated or no mutated isolates/the number of ethambutol susceptible *M. tuberculosis* isolates.
